# The diagnosis of left ventricular assist device thrombosis

**DOI:** 10.1007/s12471-015-0705-6

**Published:** 2015-06-04

**Authors:** H.Z.R. Gerds, J. Brügemann, M. Rienstra, M.E. Erasmus

**Affiliations:** Thorax Center UMC Groningen, Groningen, The Netherlands

**Keywords:** Left ventricular assist device, Heart transplantation, Pump thrombosis

## Abstract

The clinical course of a patient with a left ventricular assist device is described. A total of 6 weeks after device insertion, the lactate dehydrogenase (LDH) level increased to 2801 U/l despite adding low-molecular-weight heparin to acenocoumarol and aspirin. Pump thrombosis was suspected but unconfirmed by computed tomography. Increased pump power requirement did not occur. Instituting unfractionated heparin caused a drop in the LDH level. After discontinuing heparin, the LDH levels rose to 5529 U/l whereupon pump replacement was performed. LDH levels, combined with clinical deterioration and right heart catheterisation, led to the diagnosis of pump thrombosis.

## Introduction

Implantation of a permanent left ventricular assist device (LVAD) in a patient with cardiogenic shock must be avoided. Short-term external left ventricular support and appropriate pharmacological treatment can be a feasible alternative [1, 2]. Mechanical circulatory support by an implantable device is, however, the current therapy of choice for patients who deteriorate clinically while on the waiting list for a heart transplant [3]. Well-known complications after insertion of an LVAD are bleeding, right ventricular dysfunction, (driveline) infection, arrhythmias and pump thrombosis [4]. The incidence of pump thrombosis was reported in a range of 1.4–4 % in 2010 [5]. We report a case of early postoperative pump thrombosis.

**Fig. 1:**
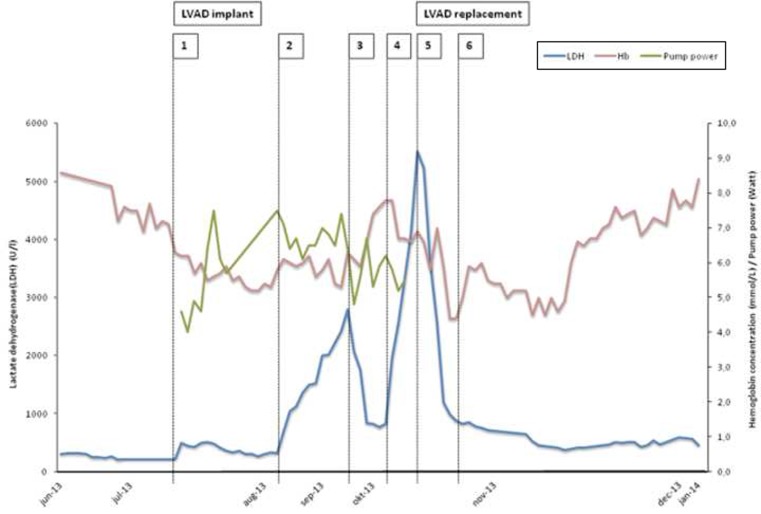
Lactate dehydrogenase (LDH) level (primary Y axis), haemoglobin (secondary Y axis) and left ventricular assist device pump power (secondary Y axis) during follow-up. Numbers 1–6 (*top of figure*) correspond with the numbers in the text

**Fig. 2:**
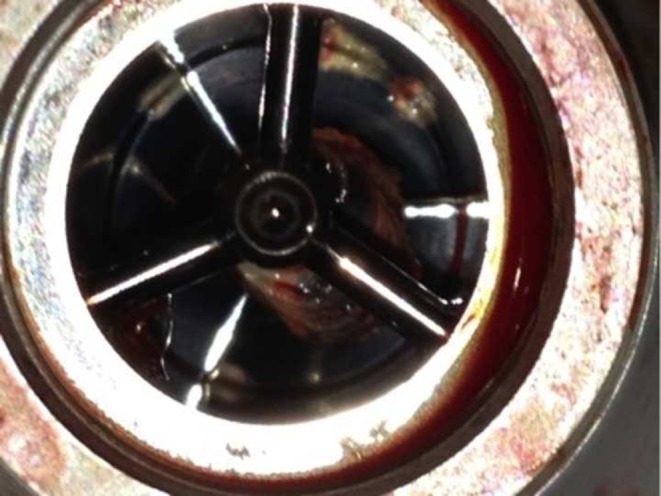
Thrombus localised on the bearing of the inlet cannula

A 46-year-old man with dilated cardiomyopathy and end-stage heart failure, despite optimal medical and implantable cardioverter defibrillator therapy, was accepted on the Eurotransplant waiting list for heart transplant on May 2013. In July 2013, due to clinical deterioration, he received—in a non-emergency setting—an LVAD type HeartMate II (Thoratec Corp. Pleasanton CA, USA) as a bridge to transplantation [Fig. 1, label 1]. Postoperative recovery was without complications, and the patient was discharged to a cardiac rehabilitation centre 19 days after insertion of the device. In all, 27 days after implantation, the level of lactate dehydrogenase (LDH) increased from 254 U/l (pre-VAD) to 704 and 1050 U/l [Fig. 1, label 2]. This raised the suspicion of pump thrombosis and low-molecular-weight heparin was added to International Normalized Ratio (INR)-guided (1.5–2.5) acenocoumarol and 100 mg of aspirin daily. However, the LDH levels further increased to 2801 U/l [Fig. 1, label 3]. The patient was hospitalised. Although computed tomography (CT) using contrast showed no signs of obstruction in the VAD circuit and LVAD power requirement did not increase [Fig. 1, label 3], unfractionated heparin (target Activated Partial Thromoplastin Time (APTT) level 40–60 s) was given intravenously. This led to a drop in the LDH level to 778 U/l [Fig. 1, label 4]. The patient was discharged, but within a few days he complained of fatigue, dyspnoea and reduced exercise tolerance. On re-admission, echocardiography showed an aortic valve opening at each contraction, which was observed more often than in previous echocardiographies. There were no signs of aortic valve regurgitation. Right ventricular function was impaired, indicated by a tricuspid annulus plane systolic excursion of 10 mm. A velocity of the tricuspid annular systolic motion s’RV of 5.3 cm/s was more impaired than earlier. Pump power requirement remained unaltered. The LDH level rose to 1967 U/l [Fig. 1, label 5]. No lung embolisms or LVAD obstructions were found by repeated contrast CT. Right heart catheterisation showed a cardiac output of 2.96 l/min, which was less than the pre-LVAD calculation of 4.45 l/min. Besides our suspicion of LVAD thrombosis, we ruled out right ventricular overload by reducing pump speed with 400 rotations/min. Sildenafil and milrinone were added to the medication in an attempt to improve right ventricular function but did not lead to clinical or biochemical improvement. In contrast, LDH rose to 5529 U/l [Fig. 1, label 6]. Since we had no other possible explanation than pump thrombosis, we replaced the LVAD next day using the subcostal approach and found a thrombus localised on the bearing of the inlet cannula (Fig. 2). Clopidogrel replaced aspirin, and the target INR level was raised to 2.5–3.5 by means of home monitoring. The patient recovered and was discharged first to the cardiac rehabilitation centre and then home. Currently (28 April 2014) the LDH level is 404 U/l.

## Discussion

Our patient demonstrates that the diagnosis of LVAD thrombus can be difficult because of discrepancies in biochemical, echocardiographic and LVAD diagnostics. First, the initially raised LDH concentrations could have also been a marker of haemolysis instead of thrombosis since the haemoglobin decreased from 7.7 to 4.4 mmol/l. Second, the pump power requirement was more or less constant in the interval between LVAD insertion and exchange. Third, subsequent echocardiography (eight recordings between first LVAD and the replacement) revealed stable findings. In particular, the frequency of aortic valve opening did not increase during follow-up, except directly before LVAD replacement. Finally, repeated contrast CTs did not support our clinical- and biochemical-based suspicion of LVAD thrombosis.

Increased power requirement of the pump can be pathognomonic for an impeller thrombus but is absent when a thrombus is localised at a HeartMate II inlet bearing ball [6]. We did not apply the suggested ramp test [7]. An unexpected, abrupt increase in incidence of LVAD thrombosis was reported recently [8]. In 2011, the incidence of confirmed pump thrombosis was 2.2 %. By January 2013, this had increased to 8.4 % (95 % confidence interval 5–13.9). In addition, the median time from implantation to thrombosis was 18.6 months, but is now much shorter, i.e. 2.7 months. Six weeks after device insertion, a sharp rise in LDH level closely suggested confirmed pump thrombosis. The exact cause of the increased rate of thrombosis remains unclear. After confirmation of the diagnosis, thrombolytic therapy might be an option [9] but pump replacement can be performed with low mortality [10]. It is crucial to monitor LDH levels, among other parameters, to track pump thrombosis in time [11] and apply the algorithm for the diagnosis and management of suspected pump thrombus [12].

In summary: in a LVAD patient receiving adequate oral anticoagulation and antiplatelet therapy despite no increase in pump power requirement, in the case of LDH increase and decrease after intravenous anticoagulation, the diagnosis of pump thrombosis located at the inlet bearing ball is highly likely. Consequently, only pump exchange is probably life-saving.

### Funding

None.

### Conflict of interest

None declared.
